# Predicting the distribution of *Stipa purpurea* across the Tibetan Plateau via the MaxEnt model

**DOI:** 10.1186/s12898-018-0165-0

**Published:** 2018-02-21

**Authors:** Baibing Ma, Jian Sun

**Affiliations:** 1 Synthesis Research Centre of Chinese Ecosystem Research Network, Key Laboratory of Ecosystem Network Observation and Modelling, Institute of Geographic Sciences and Natural Resources Research, Chinese Academy of Sciences, 11A, Datun Roadm, Chaoyang District, Beijing, 100101 China; 2 School of Earth Science and Resource, Chang’an University, Xi’an, 710000 China; 3 Department of Ecology, Evolution, and Natural Resources, Grant F. Walton Center for Remote Sensing & Spatial Analysis, School of Environmental and Biological Sciences, Rutgers University, New Brunswick, NJ 08901 USA

**Keywords:** MaxEnt, Model simulation, Species distribution, *Stipa purpurea*, Tibetan Plateau

## Abstract

**Background:**

The ecosystems across Tibetan Plateau are changing rapidly under the influence of climate warming, which has caused substantial changes in spatial and temporal environmental patterns. *Stipa purpurea*, as a dominant herbsage resource in alpine steppe, has a great influence on animal husbandry in the Tibetan Plateau. Global warming has been forecasted to continue in the future (2050s, 2070s), questioning the future distribution of *S. purpurea* and its response to climate change. The maximum entropy (MaxEnt) modeling, due to its multiple advantages (e.g. uses presence-only data, performs well with incomplete data, and requires small sample sizes and gaps), has been used to understand species environment relationships and predict species distributions across locations that have not been sampled.

**Results:**

Annual mean temperature, annual precipitation, temperature seasonality, altitude, and precipitation during the driest month, significantly affected the distribution of *S. purpurea*. Only 0.70% of the Tibetan Plateau area included a very highly suitable habitat (habitat suitability [HS] = 0.8–1.0). Highly suitable habitat (HS = 0.6–0.8), moderately suitable habitat (HS = 0.4–0.6), and unsuitable habitat (HS = 0.2–0.4) occupied 6.20, 14.30 and 22.40% of the Tibetan Plateau area, respectively, and the majority (56.40%) of the Tibetan Plateau area constituted a highly unsuitable habitat (HS = 0–0.2). In addition, the response curves of species ecological suitability simulated by generalized additive model nearly corresponded with the response curves generated by the MaxEnt model.

**Conclusions:**

At a temporal scale, the habitat suitability of *S. purpurea* tends to increase from the 1990s to 2050s, but decline from the 2050s to 2070s. At a spatial scale, the future distribution of *S. purpurea* will not exhibit sweeping changes and will remain in the central and southeastern regions of the Tibetan Plateau. These results benefit the local animal husbandry and provide evidence for establishing reasonable management practices.

**Electronic supplementary material:**

The online version of this article (10.1186/s12898-018-0165-0) contains supplementary material, which is available to authorized users.

## Background

During the last 100 years, global warming has caused substantial changes in spatial and temporal environmental patterns [1], especially in high-altitude regions [2], and these changes also determine the viability and conservation of species [1]. As the world’s third pole, Tibetan Plateau has a fragile vegetation that is vulnerable to climate change [2], and temperature and precipitation have been reported to be the main factors affecting the vegetation dynamic [3]. Furthermore, low temperature is taken as one of the most momentous limiting factors for the performance of alpine plants, whereas warming reinforces photosynthetic capacity and growth rates of these alpine plants [4, 5]. In addition, both topographic (e.g. slope, aspect, altitude and so on) and soil (e.g. soil physical, chemical and biological properties) factors play a vital role in plant growth across Tibetan Plateau [6].

*Stipa purpurea*, a perennial grass species from the family poaceae, is widespread throughout the Tibetan Plateau [7]. As a dominant herbage resource in alpine steppe of the Tibetan Plateau, the production of *S. purpurea* has a great influence on animal husbandry on the Plateau [8]. *S. purpurea* steppe constitutes a horizontal zonation in the plains and vertical zonation on mountain slopes [9, 10]. Due to its strong resistance to cold, drought and gale, *S. purpurea* can grow well in severe alpine environments, thus playing an important role in the preservation and stabilization of landscape diversity and heterogeneity [11]. Apart from preventing wind damage and regulating sand, *S. purpurea* conserves water and soil to preserve the stability of natural habitat [7]. However, *S. purpurea* as a plant species with one of the highest altitudinal distribution in the world is now suffering from global climate change and factitious disturbances [12]. The distribution of *S. purpurea* is affected not only by geographic location, but also by biological factors and natural and anthropogenic disturbances. Among abiotic disturbances, drought stress has drawn much attention because it is a main limiting factor for crop yield. The available soil water, which is largely determined by regional rainfall, affects plant growth, biomass accumulation, and leaf gas exchange rates [13].

Because of the concerns regarding the changing abiotic conditions in the Tibetan Plateau, there is an increasing interest in developing predictions to understand future communities. One tool to do this is species distribution modeling (SDM) which employs suitability indices. Suitability indices describe the relationship between habitat suitability score and a given environmental variable of a target species. Habitat suitability is a way to predict the suitability of habitat at a certain location for a given species or group of species based on their observed affinity for particular environmental conditions [14]. However, the ability to predict species distributions is highly dependent on the way in which the models are constructed, the quality, quantity, and availability of the records of true absence, and the distribution of present species, and the environmental predictor variables used to model the potential distribution of the species [15–17]. SDM depicts the relationships of different ecological variables and assesses habitat suitability for a given species. Climatic changes can affect the distribution of interacting species, which in turn may change the interactions [18]. However, species interactions may also affect climate change, for example, by changing community dynamics [19]. Understanding habitat suitability is thus critical for the development of long-term conservation strategies. Therefore, there is growing interest in understanding habitat suitability, species distributions, and habitat ecology for an improved environmental management across climates and terrestrial ecosystems [20, 21].

There are many types of typical SDMs, such as MaxEnt [22], BIOCLIM [23], DOMAIN [24], generalized additive model (GAM) [25], GLM [26], and BIOMAPPER [27], et al. We selected maximum entropy (MaxEnt) modeling because of its multiple advantages: (1) it uses presence-only data and performs well with incomplete data; and (2) requires small sample size and allows gaps [28]. Using the current distribution data for *S. purpurea* and the present climate data, the present study aimed to predict the effects of future climate change on the distribution of *S. purpurea* across the Tibetan Plateau using the MaxEnt model. The objectives of this study were to: (1) model the influence of bioclimatic and topographic factors on species distribution patterns; and (2) discuss the change in habitat suitability distribution during three periods (the 1990s, 2050s and 2070s) in the Tibetan Plateau. The results will contribute to better understanding of the processes and mechanisms of adaptation and diffusion of biology under complex climate and environmental conditions and provide theoretical basis and guidance for the management of agriculture and animal husbandry in the region.

## Methods

### Study area

The Tibetan Plateau (26°00′–39°47′N, 73°19′–104°47′ E) is located in northwestern China. It is situated at extreme altitude, with an average elevation of over 4000 m, and covers an area of approximately 2.58 million km^2^. Precipitation and temperature have clear regional distribution patterns, with annual precipitation increasing from roughly 50–700 mm from the northwest to the southeast, and annual temperature increasing gradually from − 15 to 20 °C from the northeast to the southeast [29]. The region is characterized by simultaneous heat and moisture, with a two distinct seasons, and the precipitation that diminishes from south to north and from east to west. Vegetation types from central to western Tibetan Plateau are alpine meadow, alpine steppe, alpine shrub grassland and desert grassland [30].

### Data compilation

#### *S. purpurea* data

**Fig. 1:**
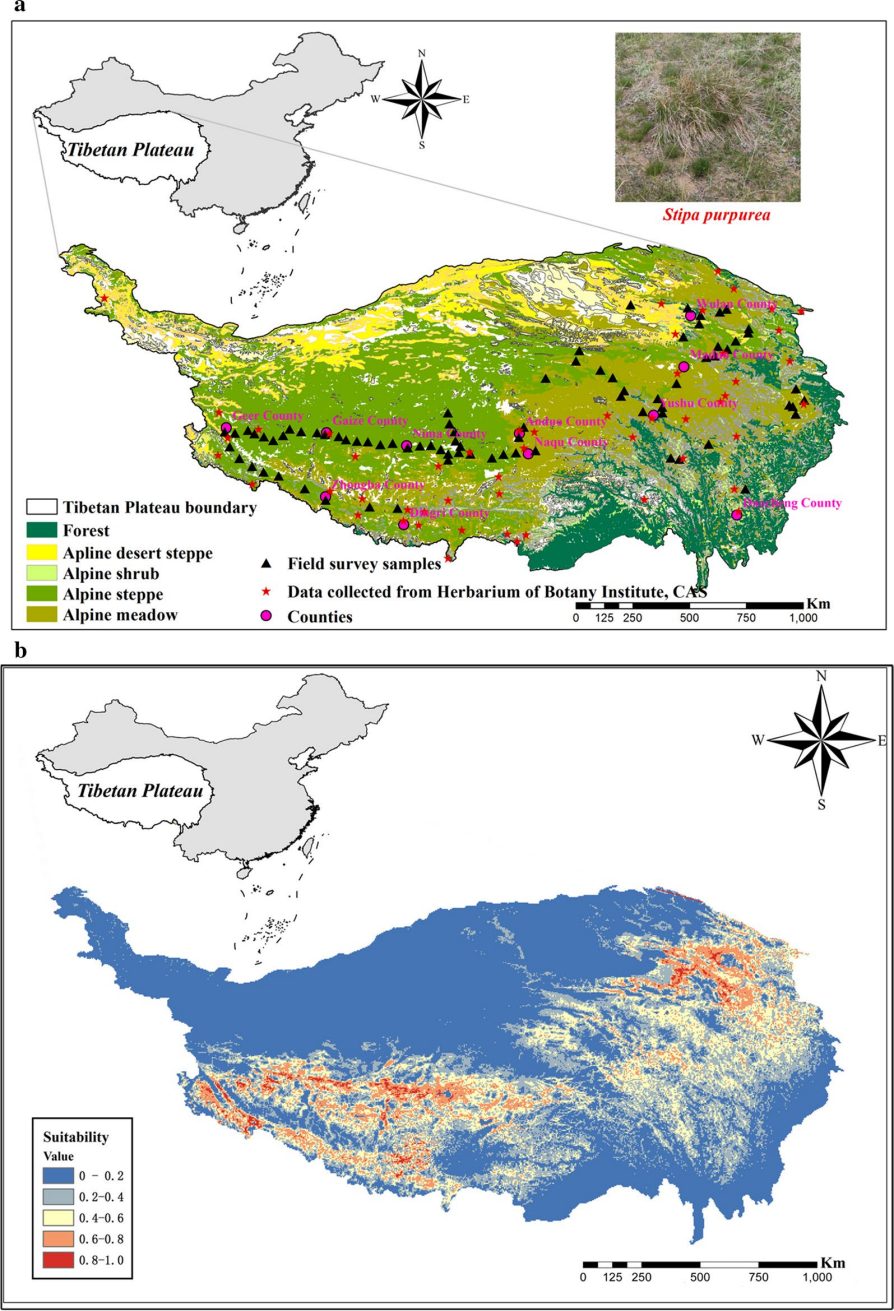
The vegetation types of grasslands in Tibetan Plateau. The distribution point and source of *S. purpurea* in Tibetan Plateau.(*S. purpurea* I represents field survey sample; *S. purpurea* II represents Herbarium of Botany Institute, CAS; The Sample collection route stands for field survey roadmap in Tibetan Plateau) (**a**). Habitat suitability distribution of *S. purpurea* (**b**)

From July to mid-August in 2015, we conducted a multisite survey during the peak growing season in 11 counties: Geer, Gaize, Nima, Zhongba, Dingri, Anduo, Nagqu, Yushu, Maduo, Wulan and Daocheng. The surveyed species indices included vegetation coverage, density, and height within each quadrat (0.5 m × 0.5 m), and the distribution of *S. purpurea* over the Tibetan Plateau was recorded from 80 specimens. Furthermore, 52 samples were obtained from the Herbarium of Botany Institute at the Chinese Academy of Sciences. Figure 1a shows the detailed distribution of *S. purpurea* in the Tibetan Plateau (Additional file 1: Table S1). The datasets used and analysed during the current study available from the corresponding author on reasonable request.

### Environmental variables

Bioclimatic variables are crucial in defining species’ environmental niches. Data for 19 bioclimatic variables were downloaded from WorldClim-Global Climate Data (www.worldclim.org/bioclim), which included the current conditions (interpolations of observed data, representative of 1960–1990) and future conditions (the 2050s, 2070s), as well as the downscaled global climate model data from CMIP5 (IPCC Fifth Assessment Report, AR5). All environmental data used in this model had a 30 arc-second spatial resolution (also referred to as 1 km spatial resolution). The topographic variables included slope, aspect and altitude. The altitude variable with 30 m spatial resolution was downloaded from Geospatial Data Cloud (http://www.gscloud.cn/). The slope and aspect variables were calculated using ArcToolbox, Spatial Analyst, Surface Analyst in GIS 10.2 (Esri, Redlands, CA, USA).

Representative concentration pathways (RCPs) are four greenhouse gas concentration (not emissions) trajectories adopted by the Intergovernmental Panel on Climate Change (IPCC) in its AR5 in 2014 [31]. This supersedes the Special Report on Emissions Scenarios (SRES) projections published in 2000 [32]. These pathways are used in climate modeling and research to describe four possible future climates, all of which are considered possible depending on how many greenhouse gases are emitted in the near future. The four RCPs—RCP2.6, RCP4.5, RCP6, and RCP8.5—are named after a possible range of Radiative Forcing values in the year 2100 relative to pre-industrial values (+ 2.6, + 4.5, + 6.0, and + 8.5 W/m^2^, respectively) [33]. Here, we selected the RCP2.6 and RCP8.5 models to simulate habitat suitability distributions of *S. purpurea* in the 2050s and 2070s. MaxEnt output for habitat suitability distribution of the species were reclassified in GIS 10.2 (Esri, USA) with ArcToolbox, Spatial Analyst and Reclassify. To compare the changes in the area of suitable habitat in RCP2.6 from the 1990s to 2050s, we used the ecological suitability index of 0.60 as the threshold to distinguish the stand or fall of the ecological suitability—values greater than 0.60 were defined as better suitability and less than 0.60 as poor suitability.

### Data analysis

**Fig. 2:**
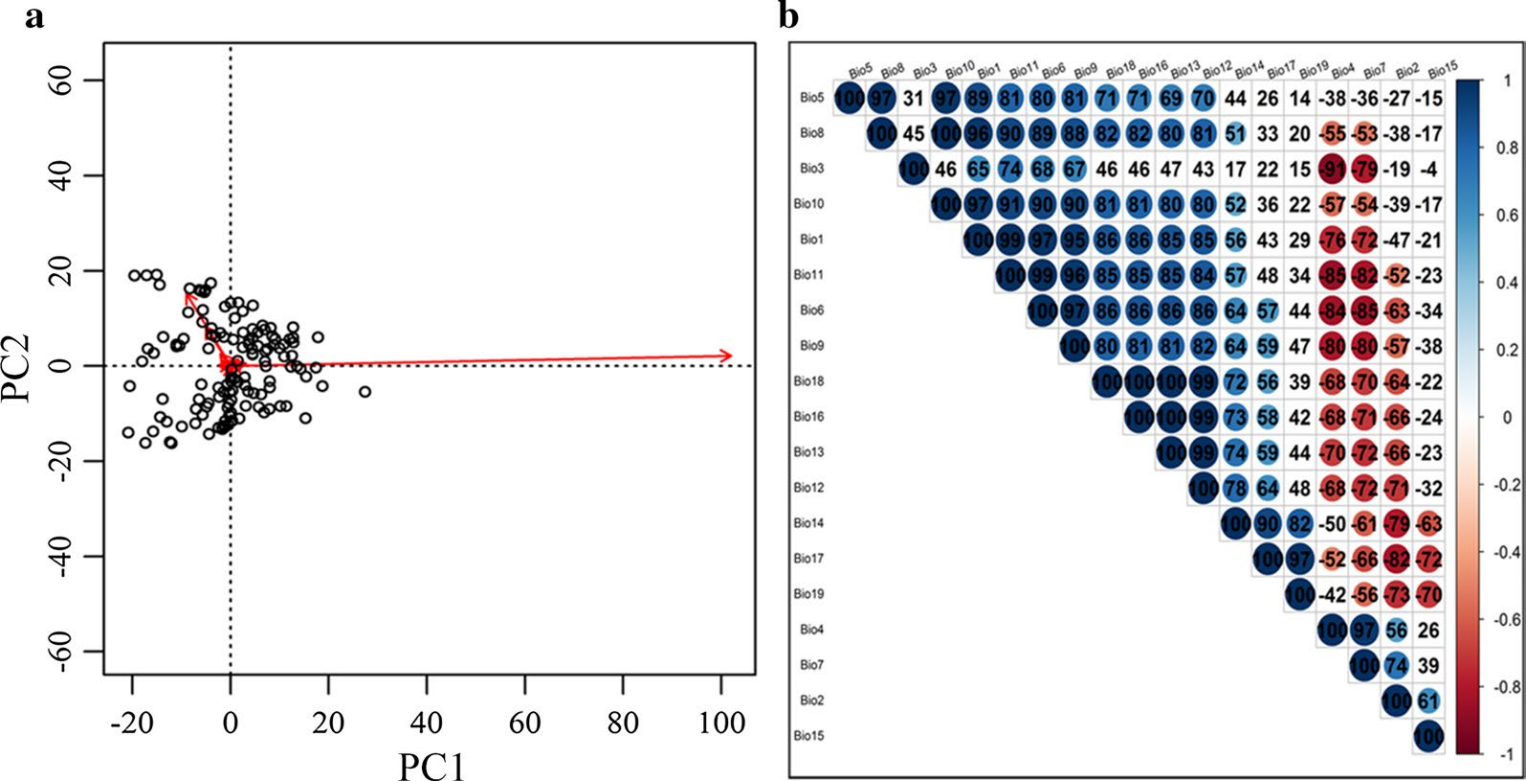
The principal component analysis (PCA) of environmental variables (**a**). Correlation analysis of the independent variables (**b**), and the colored solid circles represent the significant correlation (*P* < 0.05)

To establish a model that has better performance with fewer variables, we performed a correlation analysis and principal component analysis, and then screened eight bioclimatic variables (Table 1) to explore the response of *S. purpurea* to climate change. Only one variable from each set of highly cross-correlated variables (r^2^ > 0.85; Fig. 2) was kept for further analysis [14]. These 19 bio-climate variables were extracted from the corresponding layers using ArcGIS 10.2 (Esri, USA). In addition, topographic variables altitude, slope and aspect were chosen to explore the response of *S. purpurea* to terrain factor change (Table 1).

**Table 1:** The selected environment variables for modeling the habitat suitability distribution of *S. purpurea*

Data source	Category	Variables	Abbreviations	Units
Geospatial data cloud	Topographic	Altitude	DEM	m
		Slope	Slope	Degree
		Aspect	Aspect	Degree
WorldClim	Bioclimatic	Annual mean temperature	Bio1	°C
		Mean diurnal range (mean of monthly (max temp − min temp))	Bio2	°C
		Isothermality (BIO2/BIO7) (*100)	Bio3	–
		Temperature Seasonality (standard deviation * 100)	Bio4	°C
		Annual precipitation	Bio12	mm
		Precipitation of driest month (3 months)	Bio14	mm
		Precipitation seasonality (Coefficient of variation)	Bio15	Fraction
		Precipitation of coldest quarter (3 months)	Bio19	mm

Eight bioclimatic variables were selected from nineteen bioclimatic which downloaded from WorldClim-Global Climate Data (www.worldclim.org/bioclim). The altitude variable with 30 m spatial resolution was downloaded from Geospatial Data Cloud (http://www.gscloud.cn/). The slope and aspect variables were calculated using ArcToolbox—Spatial Analyst—Surface Analyst in GIS 10.2

The package mgcv in R language [34] was used to establish the GAM and verify the results of the MaxEnt model response curves for 11 environmental variables.

### Model simulation

#### Maximum entropy (MaxEnt) model

The MaxEnt theory was proposed by Jaynes [35]. It allows a probability distribution of maximum entropy to be calculated for the modeled target based on a set of environment variables [22].

For a random variable λ that has n different potential results X_1_, X_2_, … X_n_, for which the occurrence probabilities are P_1_, P_2_, … P_n_, respectively, the entropy of λ is given by the formula [14]:

1 $${\text{H}}\left(\uplambda \right) = \mathop \sum \limits_{{{\text{i}} = 1}}^{\text{n}} {\text{P}}_{\text{i}} { \log }\frac{1}{{{\text{P}}_{\text{i}} }} = - \mathop \sum \limits_{{{\text{i}} = 1}}^{\text{n}} {\text{P}}_{\text{i}} {\text{logP}}_{\text{i}}$$

The MaxEnt theory can be employed to predict species habitat suitability as follows. If we know nothing about local ecological conditions or a species’ life habits, the most practical prediction is that the probabilities that the area is either suitable or not for the species are both 0.5. Any data that indicate that species is present within a set of local ecological conditions is information that will reduce the uncertainty of a MaxEnt model. The more information is available, the more the uncertainty is reduced. The MaxEnt method is used to establish a model with the maximum entropy consistent with available knowledge [36]. We established the models with MaxEnt modeling version3.3.3k, which can be downloaded free from http://www.cs.princeton.edu/.

The receiver operating characteristic (ROC) describes corresponding values for omission error (FPR—horizontal axis) and sensitivity (TPR—vertical axes), with one point for each unique threshold value. The area under the ROC (AUC) was used as the MaxEnt predictive performance metric under the ROC curve. AUC values, which were obtained for both the training and evaluation data sets, are named training and testing AUC, respectively [37]. AUC values are used generally for qualitative characterization of distribution models. Araújo et al. [38] expanded a model evaluation index to “fail” for 0.50 < AUC < 0.60, “poor” for 0.60 < AUC < 0.70, “fair” 0.70 < AUC < 0.80, “good” 0.80 < AUC < 0.90, and “excellent” for 0.90 < AUC < 1.00. The AUC of the ROC plot for test localities (AUC_test_) can be used as the basis for MaxEnt model tuning of the settings [39]. Higher values reflect better ability of the model to discriminate between conditions at withheld (testing) occurrence localities and those at background localities (by ranking the former higher than the latter based on their predicted suitability values) [40].

Many recent studies have shown that the current default settings in MaxEnt were based on an extensive empirical tuning study, which can result in poorly performing models [41, 42]. For the original predictor variables (‘feature class’ or FC), allowing more FC enables more flexible and complex fits to the observed data. Users can specify which FC will be allowed, and adjust the level of regulation multiplier (RM; default = 1.0). However, higher flexibility can increase the propensity for model overfitting. We built models with RM values ranging from 0 to 4 (increments of 0.5) and with six different FC combinations (L, LQ, H, LQH, LQHP, LQHPT; where L = linear, Q = quadratic, H = hinge, P = product and T = threshold). In the present study, we used the method of collecting samples and sample information to predict the distribution of *S. purpurea*.

#### Model verification method

The flexibility of the GAM enables to predict the functional form of the appropriate variable. As an exploratory tool, the smoothing and additive models are of high value, and many environmental and ecological studies used the fitted additive model as the final model [43].

Because *S. purpurea* is affected by both biological and abiotic environmental variables, the relationship between its spatial distribution and a particular environmental variable is not always linear. Therefore, a proper parametric method should be implemented when examining the relationships between ecological suitability of *S. purpurea*’s and environmental variables.

We used GAM to verify the outputs of MaxEnt. In ArcGIS10.2 (Esri, USA), we selected transects of environmental variables and extracted each environmental variable to construct the model. The selected transects are given in the supporting information (Additional file 2: Figure S1, S2 and S3). The model can be expressed as:

2 $${\text{G}}\left( {\text{MUY}} \right) =\upalpha_{0} + {\text{f}}_{1} \left( {{\text{x}}_{1} } \right) + \cdots \cdots + {\text{f}}_{\text{n}} \left( {{\text{x}}_{\text{n}} } \right) +$$

where MUY is the expectancy value of Y, G(MUY) is copula, α_0_ is the intercept, in f_n_(x_n_), f_n_(···) is the single-variable function used to explain variable x_n_ and ε is the random variable.

Before analyzing the relationship between ecological suitability and an individual variable, the normal Q–Q should be used, and the copula can be determined based on a roughly normal distribution of ecological suitability. Here, identity link: g(z) = z was selected as the copula. Through correlation analysis and principal component analysis, we selected 11 environmental variables that are thought to be uncorrelated or have little relationship.

## Results

### Model training

#### Model performance and contribution of environmental variables

**Fig. 3:**
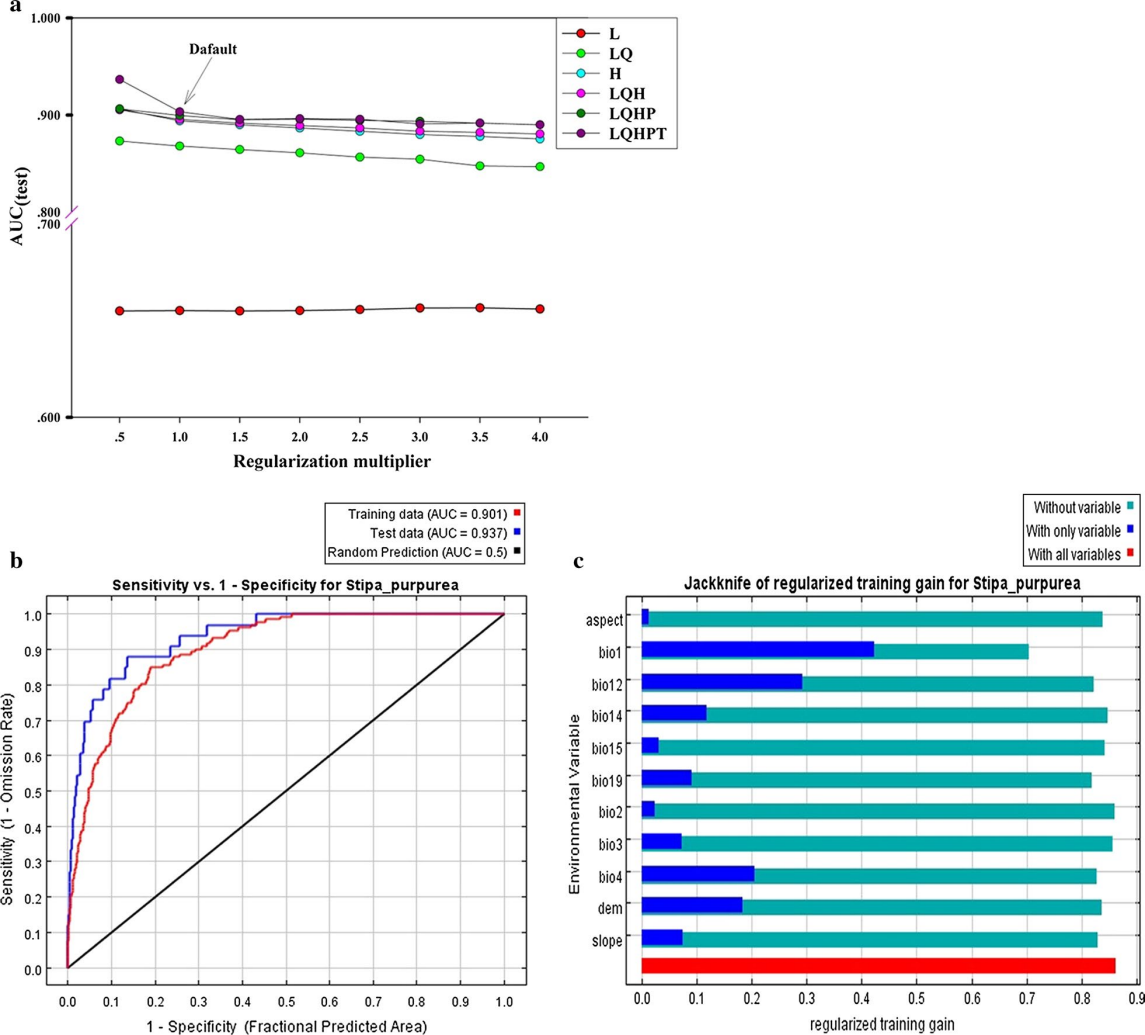
**a** Shows the value of AUC_test_ in different MaxEnt model settings (L = linear, Q = quadratic, H = hinge, P = product and T = threshold). **b** Represents the results of the AUC (area under ROC) curves in developing *S. purpurea* habitat suitability model. **c** Represents the results of the jackknife test of variables’ contribution in modeling *S. purpurea* habitat suitability distribution

The outputs of AUC_test_ were significantly different under different model settings (Fig. 3a). The variable FC showed greater difference at the equal level of RM, whereas the variable RM presented little difference at the equal level of FC. For FC = LQHPT and RM = 0.5, the value of AUC_test_ reached the maximum, indicating that MaxEnt can perform well.

The ROC estimate showed that the AUC values of the training and test data-sets were 0.901 and 0.937, respectively, which revealed a high level of accuracy in the model prediction (Fig. 3b).

The results of the jackknife test of the variables’ contribution to the model are shown in Fig. 3c. Among the 11 environmental variables used to establish the model, annual mean temperature (Bio1) and annual precipitation (Bio12) had very high weights when used independently, whereas temperature seasonality (Bio4), altitude and precipitation during the driest month (Bio14) had moderate weight when used separately, indicating that these variables could affect the habitat suitability distribution of *S. purpurea.* The other variables, including isothermality (Bio3), slope, precipitation seasonality (Bio15), mean diurnal temperature range (Bio2) and aspect, showed low weight and thus indicated poor influence on the habitat suitability distribution of *S. purpurea.*

#### Response of habitat suitability to environmental variables

**Fig. 4:**
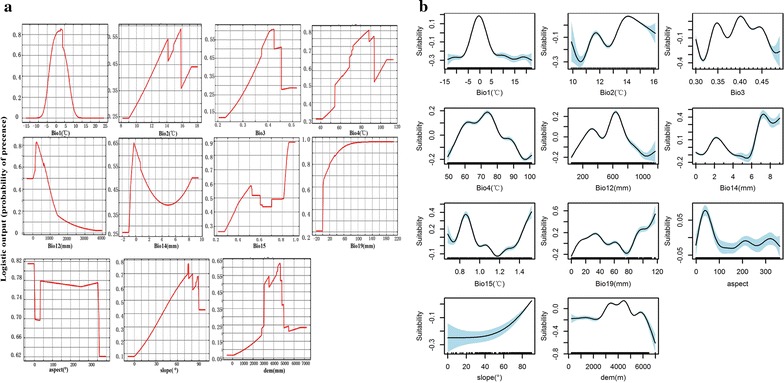
**a** Describes the response curves of 11 environmental variables in *S. purpurea* habitat distribution model. **b** Describes the MaxEnt verification results based on Generalized Additive Model (GAM)

Response curves showed the quantitative relationship between the logistic probability of the presence and environmental variables, and deepened the understanding of the ecological niche of the species by illustrating the responses of 11 variables to *S. purpurea* suitability (Fig. 4a). Based on the response curves, the suitable annual mean temperature (Bio1) ranged from − 3 to 5 °C which demonstrated that the optomal environmental temperature for growth of *S. purpurea’s* was low. The response curves of Bio2 showed that from 14 to 15 °C was the suitable annual mean temperature range. However, Bio2 did not influence the habitat suitability significantly. The optimal isothermality (Bio3), which is defined by the ratio between Bio2 and Bio7 and reflects the regional temperature fluctuation, for eco-suitability was approximately 0.40.

Greater seasonality reflects greater variability in temperature, which is illustrated by temperature seasonality Bio4 as the degree of temperature variation over a given period. The response curve for Bio4 showed that the highest probability of *S. purpurea* presence was associated with areas in where the temperature seasonality values ranged from 70 to 95. In addition, based to the response curve for precipitation during the driest month (Bio14), the suitable precipitation in the driest month was under 0 mm, which further confirmed that *S. purpurea* is drought resistant.

Based on the response curves for altitude and slope, the suitable altitude for the species is from 4200 to 4800 m at the slope greater than 70°.

#### Relationship between habitat suitability for *S. purpurea’s* and environmental Variables

GAM confirmed the relationship between the response variable (habitat/ecological suitability) and explanatory variable (environmental variable) (Fig. 4b). With respect to Bio1, the eco-suitability increased as temperature increased from − 15 to 0 °C and decreased when the temperature was greater than 0 °C. The results followed a normal distribution, with the most appropriate temperature ranging from − 3 to 3 °C. With respect to mean diurnal temperature range (Bio2), the response curve conformed approximately with the MaxEnt output. The isothermality (Bio3) in the range from 0.35 to 0.40 revealed that GAM reflected the optimal change in eco-suitability at 0.37. The ecologically suitable range of annual precipitation (Bio12) for the species was from 200 to 800 mm but it was very low as the precipitation exceeded 1000 mm. The ecological suitability declined at first and then recovered for precipitation of the direst month (Bio14). The minimum value of the precipitation seasonality (Bio15) was close to 0.70. In terms of environmental variables Bio1–Bio4, Bio12, Bio14 and Bio15, the trends of the response curves were in harmony with the outputs from the MaxEnt analysis. However, there was a significant difference in the response curve for precipitation of the coldest quarter (Bio19). The MaxEnt results showed that the eco-suitability increased with increasing Bio19.

The analysis of the response curves of the terrain variables aspect, slope, and altitude had similar curves with the modeling results of MaxEnt; the most suitable elevation for the species was estimated at 3000–5000 m. The simulations of the relationship between the response and explanatory variables using the GAM were consistent with the results obtained by MaxEnt. The AUC values of the training (0.901) and test (0.937) data-sets also indicated that the MaxEnt model was reliable in analyzing the distribution of *S. purpurea* across the Tibetan Plateau.

### Distribution and prediction of *S. purpurea*

#### Distribution of *S. purpurea*

The predicted habitat suitability was divided into five probability classes (Fig. 1b): 0–0.20 represented highly unsuitable habitat, 0.20–0.40 was unsuitable habitat, 0.40–0.60 indicated moderately suitable habitat, 0.60–0.80 was highly suitable habitat, and 0.80–1.00 indicated very highly suitable habitat. Only 0.70% of the area could be considered a very highly suitable habitat, followed by highly suitable habitat (6.20%); moderately suitable habitat (14.30%), and unsuitable habitat (22.40%). The greatest percentage (56.40%) of the Tibetan Plateau area was classified as highly unsuitable habitat. The loading of the suitability, altitude, annual mean temperature (Bio1) and annual precipitation (Bio12) layer into ArcGIS10.2 (Esri, USA) revealed that among these suitability classes, the highly suitable habitat was found only in the midlands and the northeast side of the study area, where the annual mean precipitation ranged from 0 to 899 mm, annual mean temperature was from − 3.1 to 3.8 °C,and altitude ranged from 3806 to 5654 m.

#### Predicting the distribution of *S. purpurea*

**Fig. 5:**
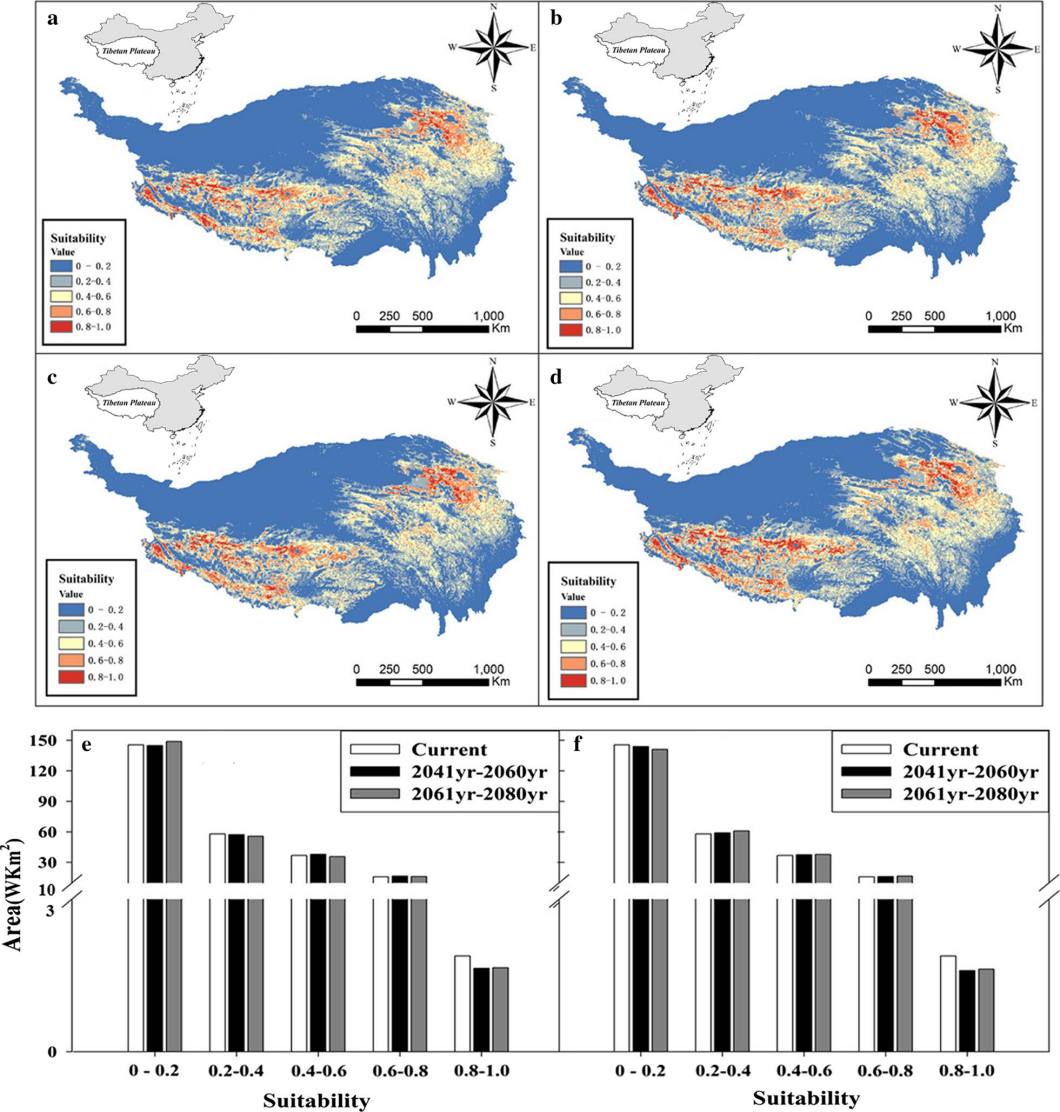
Based on MaxEnt forecast the spatial potential distribution of *S. purpurea* in 2050 s and 2070 s.(Based on RCP2.6 in 2050 s (**a**), Based on RCP2.6 in 2070 s (**b**), Based on RCP2.6 in 2050 s (**c**), Based on RCP8.5 in 2070 s (**d**). The area alteration of *S. purpurea* in current period, 2050 s and 2070 s Based on RCP2.6 (**e**); Based on RCP 8.5 (**f**)

The habitat suitability distributions of *S. purpurea’s* in the 2050s and 2070s were shown in Fig. 5a–d. Generally, the habitat suitability of *S. purpurea* in RCP2.6 and RCP8.5 revealed that habitat suitability for the species increased from the 1990s to 2050s, then decreased from the 2050s to 2070s. Figure 5e, f illustrated that the change in the distribution of *S. purpurea* under the two scenarios in the 1990s, 2050s and 2070s. The comparison of the RCP2.6 scenario between the 2050s and 1990s and between the 2070s and 1990s revealed an increase suitable area of 3739 km^2^ in the 2050s and an increase of 193 km^2^ in the 2070s, which illustrated that in the RCP2.6 scenario, the future distribution of suitable habitat for *S. purpurea* will increase with increasing temperature. The comparison of the RCP8.5 scebario between the 2050s and 1990s and between the 2070s and 1990s revealed greater amplitude of variation than that observed for RCP 2.6; an increase in suitable area with a value of 5076 km^2^ was predicted in the 2050s, but a decrease with a value of 113 km^2^ was predicted for the 2070s. This suggested that the greater change in temperature the more obvious effect could be found on the distribution of *S. purpurea*. Taken together, these results suggested that suitable habitat for *S. purpurea* will increase from the 1990s to 2050s and then decline from the 2050s to 2070s.

## Discussion

### Relationship between *S. purpurea’s* habitat suitability and environmental variables

Among the 11 environmental variables adopted in the model, annual mean temperature (Bio1) and annual precipitation (Bio12) were the most important contributors to habitat suitability distribution of *S. purpurea* as indicated by their high weighs when used independently. Hence, precipitation and temperature are important environmental factors that affect plant growth and distribution [44]. The optimal environmental temperature for growth of *S. purpurea* is low (− 3 to 5 °C), as indicated by Bio1 response curve. Some studies indicated that *S. purpurea* is a plant adapted to semiarid alpine cold meadow or alpine steppe where the mean annual temperature is approximately − 1.5 °C [45]. Rainfall is one of the most significant factors in shaping the function and structure of plants and terrestrial ecosystems on the Tibetan Plateau [13] and the suitable annual mean precipitation is at most 1000 mm for growth of *S. purpurea*. Besides, temperature seasonality (Bio4), altitude and precipitation during the driest month (Bio14) also have an effect on the habitat suitability distribution of *S. purpurea.* The response curve for precipitation seasonality showed that the greater precipitation seasonality increased the probability that *S. purpurea* was present at a location. In the coldest season, rise of precipitation increased habitat suitability for *S. purpurea*. Wang et al. [46] also found that temperature is an important climatic factor for growth of plants in semiarid or arid regions. Based on the response curves of DEM, suitable altitude for *S. purpurea* is between 4200 and 4800 m, which corresponds to the altitude range of the alpine grassland distribution from 4500 to 4800 m [45]. Continuous pressure from environmental stresses promotes plant adaptation and evolution of numerous mechanisms for survival under adverse conditions [47]. Thus, the relatively higher percentage of fats and soluble sugars in seeds at higher altitude might provide enough energy for seed germination and initial growth of seedlings, thereby enhancing the ability of seedlings to resist harsh environmental conditions [48]. Seed germination and subsequent seedling growth are directly related to population regeneration and community formation. The seeds of *S. purpurea* by raising its growth rate compensate for this competitive disadvantage, which guarantees early development and growth of *S. purpurea* in high altitude regions [49].

The other variables, such as isothermality (Bio3), slope, precipitation seasonality (Bio15), mean diurnal temperature range (Bio2) and aspect, have little influence on the habitat suitability distribution of *S. purpurea*. In terms of the effects of rainfall on plant growth, Zhang et al. [50] concluded that precipitation changes in a growing season affect the plant growth of plants and primary productivity. There was a significant difference in response curve for precipitation in the coldest season (Bio19) between MaxEnt and GAM results. According to the MaxEnt analysis, the eco-suitability increased as Bio19 increased, whereas the GAM results indicated that the eco-suitability increased and then decreased with increasing Bio19. A trend of increased overall temperature and humidity has been detected in Tibetan Plateau in recent years, which is conducive to the growth of grassland vegetation [51]. Therefore, the response curve of ecological suitability habitat and Bio19 inferred from the MaxEnt analysis reflect better the growth conditions of *S. purpurea*. Vegetation usually starts to grow when the temperature rises above 0 °C. However, a lag of 1–2 months between vegetation growth and precipitation [44] suggested that that small amounts of rain affect plant habitat suitability in the coldest season. The response curves for slope revealed that most of the sampled specimens were found on slope greater than 70°. The reason for such unusually steep slopes is that, due to high altitude of the Tibetan Plateau, the spatial resolution of 30 m used by remote sensing to obtain the slope effect could not reflect the actual terrain of the Plateau.

### Changes in distribution of *S. purpurea* in the future

From the perspective of the overall ecological suitability of *S. purpurea,* the proportions of highly suitable habitat and very highly suitable habitat are 6.20 and 0.70%, respectively. Global warming promotes vegetation growth [5, 52, 53], and a simulation of the warming climate in the alpine meadow area of the Tibetan Plateau revealed that temperature had a positive effect on alpine steppe by accelerating the process of alpine phenology and prolonging the growing season. However, a continuous rise in temperature had a negative effect on vegetation [54, 55]). In the RCP8.5 scenario, the suitability of the overall landscape and the areas with habitats suitable for survival of *S. purpurea’* was higher than that in RCP2.6. *S. purpurea* is distributed in the central and southeastern regions of the Tibetan Plateau, and from the large spatial scale perspective, the future distribution of *S. purpurea* will not exhibit sweeping changes. Future growth conditions of *S. purpurea* will have a great impact on the livestock husbandry of Tibetan Plateau and on the modulation and improvement of the ecosystems on Tibetan Plateau and even Eurasian continent [13].

Apart from the above environmental factors, some other factors could also influence plant suitable habitats. Duan et al. [56] reported that overgrazing and excessive reclamation accelerate large-scale grassland degradation on the Tibetan Plateau. Significant decrease in grassland productivity and biological diversity has becoming a great obstacle to sustainable social economic and ecological development. Human activities including livestock overgrazing, yak trampling, and sod removal for construction, have created huge areas of “black soil” (also known as black soil-type degraded grassland). The sod layer, which is from 10 to 15 cm deep, is totally removed by intensive grazing and activities of rodents leaving the sub-soil uncovered [57]. In addition, unsustainable land use practices have resulted in great reduction of soil organic carbon and a rapid decline of soil fertility and crop productivity [58]. Once degraded, these eco-systems cannot be restored easily [59]. Therefore, our future study will focus on the effects of human activities on the distribution of *S. purpurea*.

## Conclusions

Estimating how the future distribution of *S. purpurea* will respond to rapid environmental climatic modifications is of vital importance for determining the viability and conservation of *S. purpurea*. The results indicated that on a temporal scale, the suitable habitat for *S. purpure* tend to increase from the 1990s to 2050s and then decline from the 2050s to 2070s. On a spatial scale, the future distribution of *S. purpurea* will not experience sweeping changes, and the main distribution areas of the species will remain in the central and southeastern regions of the Tibetan Plateau. Our results will benefit the local animal husbandry and provide evidence for establishing reasonable management practices.

## Additional files

**Additional file 1: Table S1.** Description of the sampled sites (*Stipa purpurea*) across Tibetan Plateau.

**Additional file 2.** The belt transects of environmental variables were chosen to construct GAM model. **Figure S1.** The belt transects of bioclimatic variables including annual mean temperature (Bio1), mean diurnal range (Bio2), isothermality (Bio3) and temperature seasonality (Bio4), respectively. **Figure S2.** The belt transects of bioclimatic variables including annual precipitation (Bio12), precipitation of driest month (Bio14), precipitation seasonality (Bio15) and precipitation of coldest quarter (Bio19), respectively. **Figure S3.** The belt transects of topographic variables including aspect, slope and DEM, respectively.

## Abbreviations

RCPs: representative concentration pathways
MaxEnt: maximum entropy
SDMS: species distribution models
ROC: receiver operating characteristic
Bio1: annual mean temperature
Bio2: mean diurnal range (mean of monthly (max temp − min temp))
Bio3: isothermality ([BIO2/BIO7] × 100)
Bio4: temperature seasonality (standard deviation × 100)
Bio12: annual precipitation
Bio14: precipitation in the driest month
Bio15: precipitation seasonality (Coefficient of variation)
Bio19: precipitation in the coldest season
