# Supplementary material for: Predicting the distribution of Stipa purpurea across the Tibetan Plateau via the MaxEnt model
Source: BMC Ecol. 2018 Feb 21;18:10. doi: 10.1186/s12898-018-0165-0 (PMC5822641; doi:10.1186/s12898-018-0165-0)
Supplement: Supplementary file 1 — Additional file 1: Table S1. Description of the sampled sites (Stipa purpurea) across Tibetan Plateau. [file 12898_2018_165_MOESM1_ESM.docx]

**Table S1** Description of the sampled sites (*Stipa purpurea*) across Tibetan Plateau

| Site | Longitude | Latitude | Altitude(m) | AMP(mm) | AMT(^o^C) |
| --- | --- | --- | --- | --- | --- |
| 1 | 80°01′39" | 32°21′17" | 4643 | 120.8 | 4.5 |
| 2 | 80°25′53" | 32°18′23" | 4546 | 126.4 | 4.4 |
| 3 | 80°13′17" | 31°46′27" | 4749 | 140.2 | 3.9 |
| 4 | 80°36′32" | 31°18′23" | 4788 | 162.8 | 3.4 |
| 5 | 81°06′35" | 30°59′02" | 4728 | 183.3 | 3.1 |
| 6 | 81°33′43" | 30°46′27" | 4593 | 202.3 | 2.9 |
| 7 | 82°09′35" | 30°36′46" | 4766 | 224.9 | 2.8 |
| 8 | 83°11′36" | 30°06′46" | 4580 | 268.6 | 3.1 |
| 9 | 84°01′02" | 29°39′40" | 4569 | 309.1 | 3.2 |
| 10 | 85°46′40" | 29°23′14" | 5342 | 353.1 | 3.4 |
| 11 | 86°52′34" | 29°20′20" | 4642 | 363.5 | 3.6 |
| 12 | 97°40′54" | 31°19′21" | 4505 | 617.7 | 5.2 |
| 13 | 98°01′15" | 31°16′27" | 4207 | 597.7 | 4.9 |
| 14 | 99°11′02" | 31°52′15" | 4770 | 580.4 | 4.0 |
| 15 | 100°38′15" | 30°05′48" | 3989 | 758.0 | 8.0 |
| 16 | 102°37′27" | 33°03′52" | 3471 | 666.4 | 6.2 |
| 17 | 102°30′40" | 33°21′17" | 3439 | 640.0 | 5.5 |
| 18 | 102°21′57" | 33°25′10" | 3443 | 638.4 | 5.2 |
| 19 | 102°57′48" | 33°36′46" | 3543 | 616.8 | 5.9 |
| 20 | 102°38′25" | 34°03′52" | 3804 | 572.1 | 5.1 |
| 21 | 94°54′13" | 34°48′23" | 4767 | 308.7 | 1.1 |
| 22 | 95°23′17" | 34°30′00" | 4513 | 343.5 | 1.0 |
| 23 | 95°50′25" | 33°59′02" | 4266 | 388.8 | 1.3 |
| 24 | 95°42′40" | 33°46′27" | 4319 | 407.9 | 1.4 |
| 25 | 96°35′01" | 33°09′41" | 4227 | 487.0 | 1.7 |
| 26 | 97°21′31" | 33°06′46" | 4366 | 486.9 | 2.3 |
| 27 | 97°20′33" | 33°20′19" | 4269 | 478.5 | 2.0 |
| 28 | 97°09′53" | 33°40′39" | 4458 | 444.2 | 1.8 |
| 29 | 97°54′28" | 34°16′27" | 4622 | 421.5 | 1.8 |
| 30 | 99°05′13" | 35°18′23" | 4121 | 394.6 | 1.5 |
| 31 | 99°25′34" | 35°22′15" | 4047 | 401.3 | 1.7 |
| 32 | 99°33′19" | 35°44′31" | 3725 | 387.9 | 1.7 |
| 33 | 99°55′37" | 35°37′45" | 3372 | 414.5 | 1.8 |
| 34 | 100°14′59" | 35°58′04" | 3145 | 408.5 | 2.2 |
| 35 | 100°45′02" | 36°17′25" | 2929 | 403.5 | 2.5 |
| 36 | 100°46′01" | 36°27′06" | 3776 | 374.4 | 2.7 |
| 37 | 99°53′40" | 37°13′33" | 3200 | 306.9 | 3.3 |
| 38 | 99°37′12" | 37°06′46" | 3211 | 320.5 | 2.5 |
| 39 | 98°48′44" | 36°38′43" | 3735 | 306.3 | 2.1 |
| 40 | 98°51′39" | 36°59′02" | 3311 | 288.2 | 2.4 |
| 41 | 98°21′36" | 37°16′27" | 3699 | 248.8 | 2.7 |
| 42 | 96°05′56" | 37°24′12" | 3512 | 156.4 | 3.2 |
| 43 | 94°03′49" | 35°34′50" | 4714 | 229.6 | 1.1 |
| 44 | 93°56′04" | 35°09′41" | 4454 | 263.6 | 0.8 |
| 45 | 92°44′21" | 34°29′02" | 4557 | 289.0 | 0.0 |
| 46 | 91°52′01" | 32°32′54" | 5268 | 403.8 | 0.2 |
| 47 | 91°40′23" | 32°08′43" | 4809 | 419.6 | 0.8 |
| 48 | 92°20′07" | 31°36′46" | 4537 | 469.6 | 1.5 |
| 49 | 91°33′36" | 31°31′56" | 4524 | 438.3 | 1.5 |
| 50 | 91°03′34" | 31°28′04" | 4627 | 405.1 | 1.3 |
| 51 | 90°36′26" | 31°19′21" | 4549 | 395.5 | 1.3 |
| 52 | 89°46′02" | 31°29′02" | 4586 | 361.7 | 1.6 |
| 53 | 89°17′56" | 31°32′54" | 4587 | 337.7 | 2.6 |
| 54 | 89°21′48" | 31°47′25" | 4553 | 335.0 | 1.8 |
| 55 | 89°10′11" | 32°00′01" | 4566 | 324.4 | 1.7 |
| 56 | 89°08′14" | 32°22′15" | 4707 | 303.6 | 1.8 |
| 57 | 88°55′38" | 32°40′39" | 4896 | 285.8 | 1.5 |
| 58 | 88°51′46" | 33°06′46" | 4853 | 262.5 | 1.6 |
| 59 | 88°52′44" | 31°14′31" | 4905 | 335.8 | 2.8 |
| 60 | 88°52′34" | 31°29′02" | 4624 | 329.9 | 2.6 |
| 61 | 88°35′17" | 31°39′41" | 4842 | 317.9 | 2.6 |
| 62 | 88°12′02" | 31°49′21" | 4540 | 300.2 | 2.7 |
| 63 | 87°45′52" | 31°48′22" | 4703 | 292.1 | 2.7 |
| 64 | 87°16′47" | 31°43′33" | 4671 | 285.8 | 2.8 |
| 65 | 86°40′56" | 31°54′12" | 4777 | 267.2 | 3.0 |
| 66 | 86°11′51" | 31°51′17" | 4774 | 246.6 | 3.6 |
| 67 | 85°41′49" | 31°58′04" | 4977 | 237.1 | 3.7 |
| 68 | 85°15′39" | 31°59′02" | 4961 | 231.6 | 3.8 |
| 69 | 84°49′29" | 32°02′54" | 4512 | 219.6 | 3.9 |
| 70 | 84°32′02" | 32°10′39" | 4582 | 209.5 | 3.7 |
| 71 | 84°00′03" | 32°15′29" | 4625 | 192.5 | 4.6 |
| 72 | 83°40′40" | 32°19′21" | 4834 | 183.4 | 4.6 |
| 73 | 83°02′53" | 32°22′15" | 4669 | 165.1 | 4.9 |
| 74 | 82°35′45" | 32°28′04" | 4449 | 150.5 | 5.0 |
| 75 | 82°20′14" | 32°12′35" | 4478 | 163.8 | 4.4 |
| 76 | 81°56′59" | 32°00′58" | 4610 | 163.9 | 4.3 |
| 77 | 81°34′41" | 32°09′41" | 5234 | 151.5 | 4.3 |
| 78 | 81°12′24" | 32°17′25" | 4526 | 143.7 | 4.3 |
| 79 | 80°56′54" | 32°25′10" | 5181 | 131.3 | 4.3 |
| 80 | 98°10′57" | 36°07′45" | 3425 | 313.2 | 1.5 |

Note. AMT= annual mean temperature; AMP = annual mean precipitation.

Data availability statement: The datasets used and analysed during the current study available from the corresponding author on reasonable request.
