# Supplementary material for: Predicting the distribution of Stipa purpurea across the Tibetan Plateau via the MaxEnt model
Source: BMC Ecol. 2018 Feb 21;18:10. doi: 10.1186/s12898-018-0165-0 (PMC5822641; doi:10.1186/s12898-018-0165-0)
Supplement: Supplementary file 2 — Additional file 2. The belt transects of environmental variables were chosen to construct GAM model. Figure S1. The belt transects of bioclimatic variables including annual mean temperature (Bio1), mean diurnal range (Bio2), isothermality (Bio3) and temperature seasonality (Bio4), respectively. Figure S2. The belt transects of bioclimatic variables including annual precipitation (Bio12), precipitation of driest month (Bio14), precipitation seasonality (Bio15) and precipitation of coldest quarter (Bio19), respectively. Figure S3. The belt transects of topographic variables including aspect, slope and DEM, respectively. [file 12898_2018_165_MOESM2_ESM.doc]

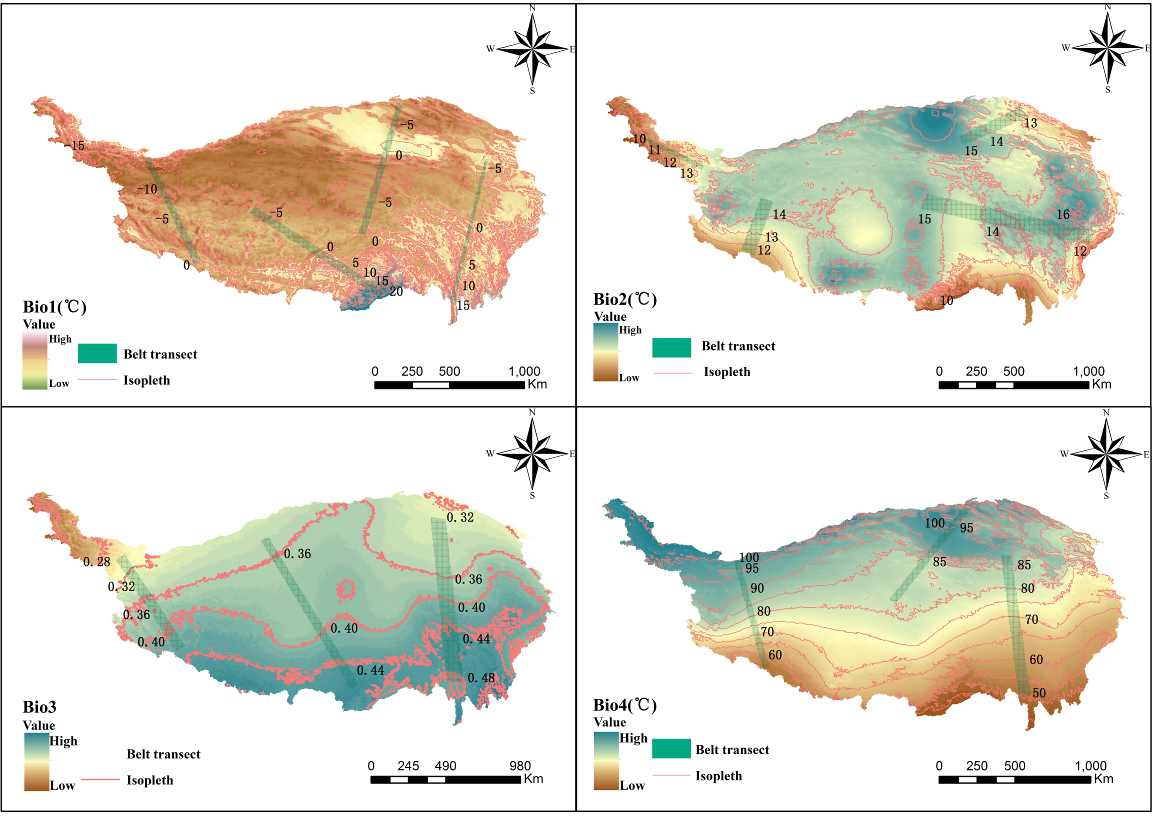


**Figure S1** The belt transects of bioclimatic variables including annual mean temperature (Bio1)，mean diurnal range (Bio2)，isothermality (Bio3) and temperature seasonality (Bio4), respectively.


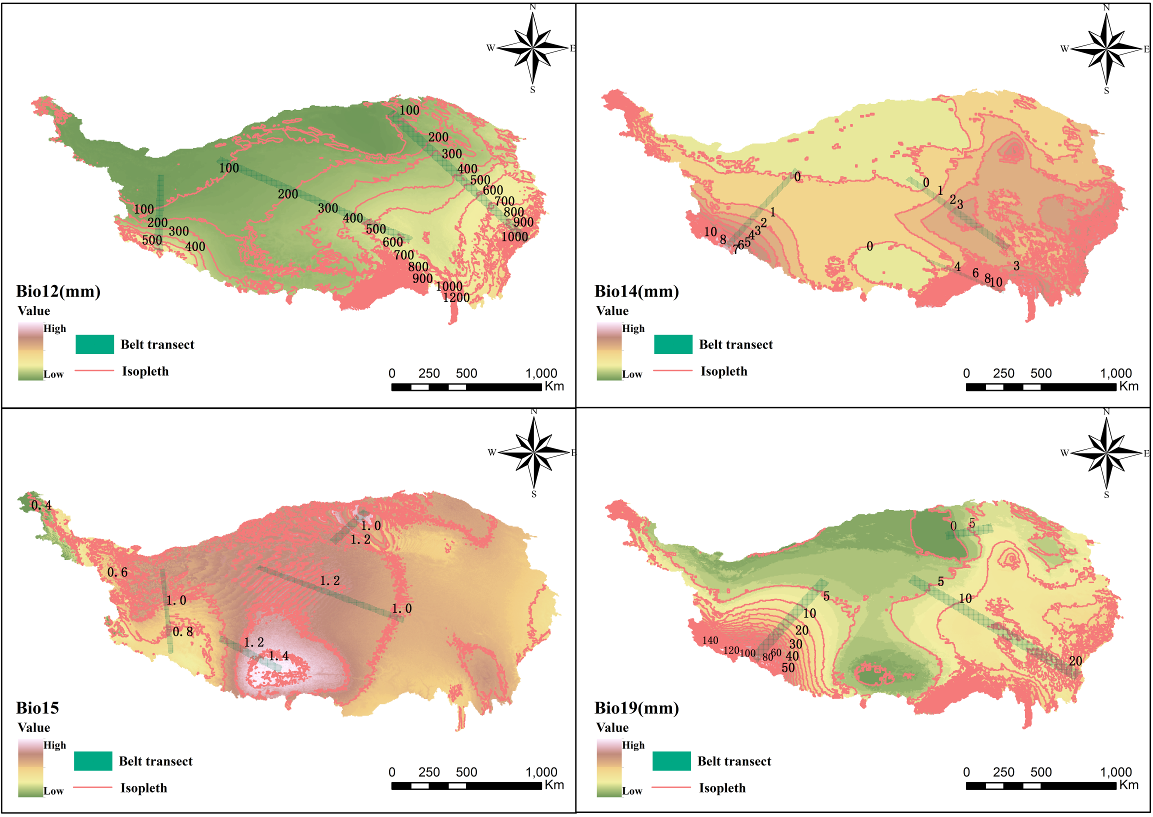


**Figure S2** The belt transects of bioclimatic variables including annual precipitation (Bio12)，precipitation of driest month (Bio14)，precipitation seasonality (Bio15) and precipitation of coldest quarter (Bio19), respectively.


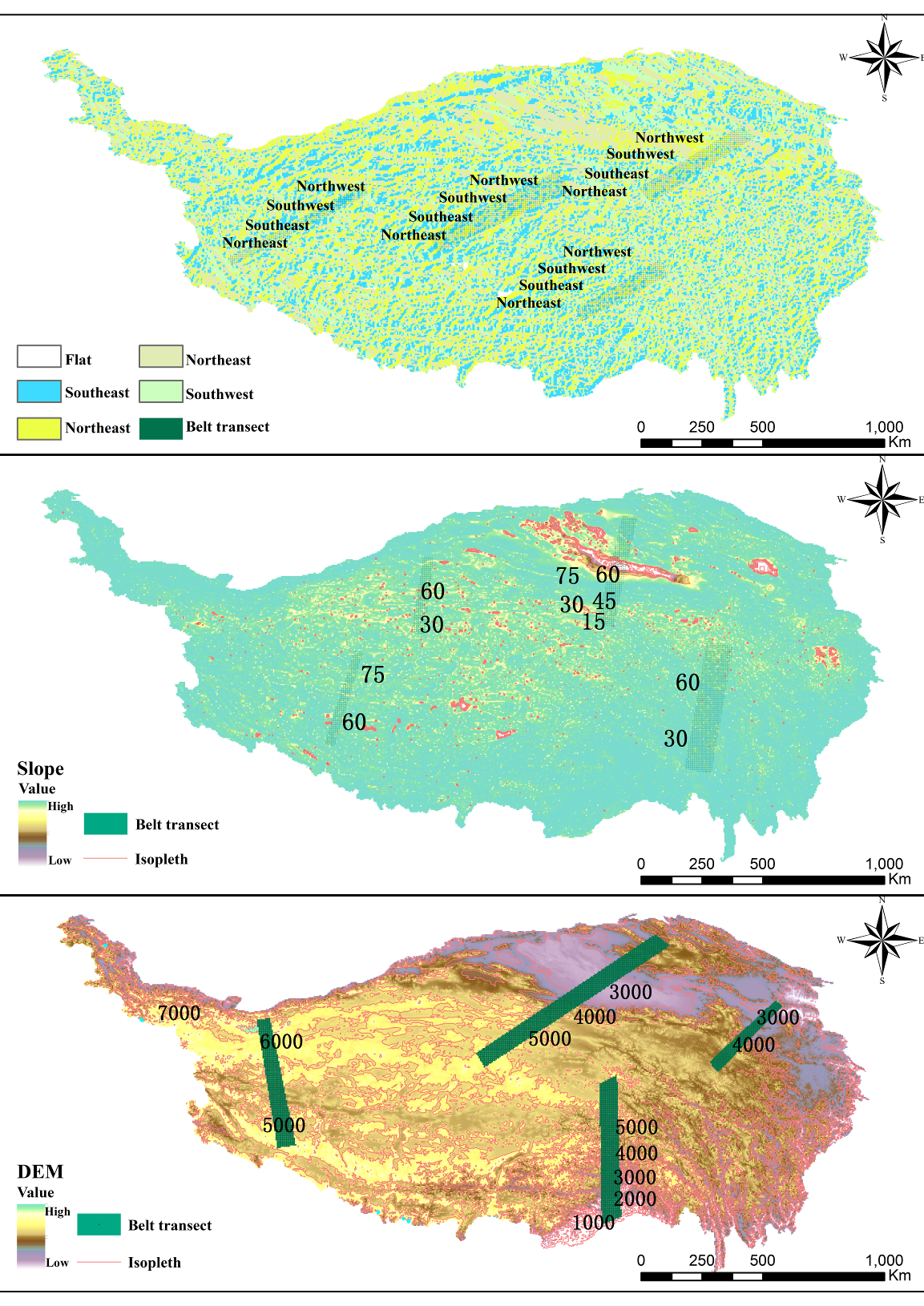


**Figure S3** The belt transects of topographic variables including aspect，slope and DEM, respectively.
